# Different training routes to qualify in occupational medicine

**DOI:** 10.3325/cmj.2019.60.560

**Published:** 2019-12

**Authors:** Luca Cegolon

**Affiliations:** 1 Local Health Unit N.2, Public Health Department, Treviso, Veneto Region, Italy; 2 University of Pavia, Master in Occupational Medicine, Pavia, Italy *l.cegolon@gmail.com*

## BACKGROUND

Occupational health aims to protect the health of workers in the worksite. Across Europe it is conducted by medical doctors qualified through various postgraduate routes, after developing the training portfolio expected from an occupational health consultant (OHC) (1-4).

An Italian law, the Legislative Decree 81/2008 (D.Lgs 81/2008) reformed the health and safety at work (HSW) system in Italy, defining also competencies and different paths to qualify as OHC in the country and establishing a controversial training curriculum, whose definition required extensive negotiations between different medical unions and various Italian governments over the years (3-8).

With the promulgation of the D.Lgs. 81/2008 (Art. 38), doctors eligible to practice as OHC in Italy are now:

- occupational medicine specialists (OMS) with postgraduate academic specialty in occupational medicine;

- physicians already qualified as OHC by amnesty before April 2008, including also doctors without any postgraduate qualification in occupational medicine (Legislative Decree 277/1991);

- academic teachers of a number of related disciplines (occupational medicine, industrial toxicology, industrial hygiene, preventive medicine for workers, labor physiology/hygiene, other), although it is not clearly specified if this allowance is restricted just to academic clinical staff or also to external experts contracted as educators by Italian universities;

- doctors with four-year experience of clinical activity “*in the field of labour*” for the Italian armed forces (including police, military police, and Finance Guard), with a vague definition of the clinical background and competences expected from these doctors;

- consultants in public health medicine and in legal/forensic medicine, conditional on the completion of an integrative training (a one-year master’s course in occupational medicine planned after the D.Lgs 81/2008). This master’s course must be organized only by Italian universities with departments in occupational medicine and with professors and academic educators in that discipline.

## DISCUSSION

The new Italian legislation on HSW (D.Lgs. 81/2008) warrants a minimum level of skills and professional training to support the tasks of an OHC, and “i*s clearly in contrast with attempts to justify training routes not recognized by the European Union as well as exceptions and amnesties of any kind for doctors without the qualifications required by the D.Lgs. 81/2008*” (3-8).

However, the time and modes of application of the D.Lgs. 81/2008 (4-8) raise many questions and are still open to various criticisms. First of all, this law blatantly damaged trainee doctors who at the time of its promulgation had chosen to specialize in public health or legal/forensic medicine with the option to practice as OHC upon completion of their postgraduate specialist medical training (4-8).

Using a concrete approach, let us consider the following “dyscrasias:”

- **Case 1**: a postgraduate trainee in public health or legal/forensic medicine in his or her last year of training in 2008 would have qualified a few months after the promulgation of the D.Lgs. 81/2008 (issued in April 2008). However, (s)he was among those doctors affected by the retroactive effects of the law and was required to undertake the integrative master’s course in occupational medicine;

- **Case 2:** a doctor completing his or her specialist training in public health or legal/forensic medicine in 2007, before the promulgation of the D. Lgs. 81/2008, would be exempt from the retroactive effects of the D. Lgs. 81/2008;

- **Case 3:** a consultant in public health or legal/forensic medicine, qualified as OHC outside academic specialist routes who took a career break for any reason during the three years preceding the promulgation of the D.Lgs. 81/2008 was among those required to undertake the integrative master’s course in occupational medicine. The same fate would not have affected either an OMS or (inexplicably) an OHC qualified by amnesty (Legislative Decree 277/1991), both taking a similar career break.

The discriminations are a matter of fact, since only some categories of doctors have been stripped of previously acquired professional rights. This legal procedure is unprecedented in Italy, considering also what happened earlier to Italian dentists and family physicians after the introduction of more restrictive professional laws in these two medical branches (3,4,9). In particular, and different from what occurred to consultants in public health medicine and legal/forensic medicine, dentists and family physicians in Italy were not subject to the retroactive application of a new more restrictive law. Only doctors qualified or registered after the introduction of new laws faced professional restrictions, whereas the others were allowed to continue working under their previously acquired rights, both within Italy and in any other EU member state (4).

The way the D.Lgs 81/2008 was introduced and applied had serious consequences from economic perspectives. Unjust damage was in fact inflicted upon consultants and trainees in public health and legal/forensic medicine. Four years elapsed before the first master’s course in occupational medicine was established in Italy. The long and unjustifiable delay in setting up these training programs prevented a number of doctors from practicing occupational medicine for a considerable period of time, creating not only professional damage but also a very substantial loss of income. This should be considered a subject for potential compensation claims. It was reasonable to expect the Italian Ministry of University, Education and Scientific Research (MIUR, Italian acronym) and the National University Council (CUN, Italian acronym) to plan and organize these master’s courses in one year. These postgraduate programs were instead set up 4 years after the promulgation of the D. Lgs 81/2008. This 4-year delay meant at least 2-3 years of economic losses in terms of missing income for each doctor affected by the legally questionable retroactive effects of the D.Lgs 81/2008 and/or the factual inefficiency of some Italian Institutions (CUN and MIUR). Consultants in public health medicine will have certainly suffered these drawbacks even more than consultants in legal/forensic medicine, since the former work primarily in the public sector, and in time of financial crisis and job cuts in the public administration this employment option has diminished significantly since 2008. Several doctors had to enroll in master’s degrees in occupational medicine very often far from home, in a limited number of universities in other regions fortunately open to these training programs. This inevitably had logistic and, above all, further economic implications, especially for the doctors residing in Northern Italy, whose only option to qualify as OHC nearby was to undertake the integrative training in occupational medicine at the University of Pavia (4).

A critical point worthy of analysis regards the competences expected and factually possessed by doctors rather than just academic titles and union affiliations (3). In fact, occupational medicine is increasingly more important nowadays and has considerably evolved over time (4,10,11). There is a clear commonality between the postgraduate specialty training curriculum in public health medicine, occupational medicine, and legal/forensic medicine in Italy. The filling of curricular gaps, with the acquisitions of the necessary outstanding professional skills, should allow a medical specialist to shift from one medical branch to another “*sister*” medical discipline, contributing a more holistic vision of postgraduate medical education (4). The case of OMS employed in the public administration and covering positions traditionally preferential for public health consultants (eg, hospital management, control of communicable diseases, and medical epidemiology) is well known. These OMS are not required to take additional training to perfectly match the professional profile of public health consultants, whose background includes more strongly recognized competencies in various important health fields: health care management, health protection, health promotion, and epidemiology.

Continuous medical education (ECM, Italian acronym) is mandatory for all practicing medical doctors in Italy (12). However, this obligation is inexplicably far more stringent for occupational medicine than for all other medical disciplines in Italy. If an OHC fails to obtain the necessary annual educational credits, (s)he faces the definite risk of removal from the Italian National Register for OHC (8,13), whereas it is unclear what happens to doctors practicing in other medical specialties. This may be related to the important economic interests surrounding occupational medicine, which could generate an “understandable” corporate obstructionism (4). In this view, the lack of publications pointing out the issues discussed above needs reflection, considering the real interest of young doctors to pursue this type of work opportunity. A possible explanation may lay in the role of some Italian medical journals, whose editorial board members include Italian academics in occupational medicine and/or fellows of the Italian Society of Occupational Medicine and Industrial Hygiene (SMLII, Italian acronym).

A further point of the D.Lgs 81/2008 regarding the eligibility criteria to practice occupational medicine is questionable. As mentioned above, in addition to OMS, an academic educator of occupational medicine or related disciplines is eligible to freely practice as OHC in Italy (8). This seems to be a strong assumption, since academic and practical clinical medicine require distinct competences often (but not necessarily) coexisting in the same professional. It is well known, for instance, that many professors of occupational medicine have recently been removed from the national registry of OHC for failure to accumulate the necessary annual ECM credits. Not to mention that quite a few professors/academics of occupational medicine are not necessarily medically qualified (in Italy and elsewhere).

An additional critical point concerns a legal dispute inside the EU. Despite the recognized different legal routes to qualify as OHC (in Italy as in several other European countries), there is still a clear disparity between Italian OMS and OHC qualified outside the academic specialty training in occupational medicine (ie, non-OMS) in terms of international recognition of their professional status across the EU member states (3,4). The European directives concern reciprocal and mutual recognition of training curricula across the EU, but it is up to the government of every single member state to negotiate with the EU and determine what would be an acceptable national training to be defended, recognized, and exported internationally (2,4). Since OMS and non-OMS have exactly the same professional status after qualifying as OHC in Italy, both in the public and private sector, an integrative legislation is clearly required to allow the issuance of a Certification of Completion of Training (CCT) also to doctors legally qualified as OHC without academic specialty training, to be used abroad and obtain a license to practice occupational medicine within the entire EU (4). Why would an Italian OHC qualified without academic specialty training in occupational medicine (non-OMS) be permitted to practice only in Italy (a full member of the EU) but not in other EU member states? What is the rationale for this discriminatory national regulation? This is a domestic unreasonable dispute, and it is up to the Italian government to put an end to this perplexing and ongoing controversy inside the EU.

The current unusual situation may be illustrated by the following, indisputable example: an Irish GP qualified in Ireland as OHC without academic specialty training could potentially practice occupational medicine in Italy by EU law, whereas the opposite is not contemplated: an Italian OHC qualified outside university specialty training routes (non-OMS) would not be allowed to practice occupational medicine, say, in Ireland (4,14,15).

## CONCLUSIONS

Union barriers and labels in the medical professions hinder substantial and constructive career development approaches based upon common sense (4,16). Most discussions on this topic have so far been helpful but seem incomplete without some crucial legal and historical reasons worthy of consideration, such as the violations and unconstitutional aspects in the way the D.Lgs 81/2008 has been applied (Art. 3 of Italian Constitution and the principle of equality before the Law) (3,4). Let us imagine a case in point that could be well epitomized by the arguments proposed: a public health trainee, in his last year of training, falls a guiltless and helpless victim of a vexatious law. In fact, the D.Lgs 81/2008 was issued just after the tragic fire of Thyssenkrupp of Turin in December 2007, which in a dramatic way should have provided some medical unions with very persuasive arguments to negotiate the legislative revisions to be included in the D.Lgs. 81/2008 with the Italian government (17-19).

It is difficult to estimate the number of doctors affected by the retroactive detrimental effects of the D.Lgs 81/2008, especially since several public health and legal/forensic medicine consultants have pursued employment options other than occupational health as a result of the above mentioned professional restrictions. A national survey in this respect may help to provide some relevant figures on this matter.

The inconclusive and up to now unanswered questions are as follows:

- Why did the professional, economic, and ethical damage inflicted to the above mentioned categories of specialist doctors (consultants in public health and legal/forensic medicine), which was submitted to the attention of Italian Administrative Courts and the Italian Council of State years ago, fall on deaf ears (20-23)?

- Who opposed and slighted these legal actions, which initially only asked for recognition of professional acquired rights, not compensation, to prevent the economic damage that subsequently and inevitably would have occurred (20-23)?

These questions obviously involve primarily law makers, whose decisions need to be opposed when they have massive detrimental effects on personal and professional lives of individuals. A clear and decisive stance from the Italian medical associations and CUN on this matter would have been welcome.

Beyond compensation claims, the most immediate and easier integrative revision to be introduced in the D.Lgs 81/2008 would certainly be the issuance of a CCT for all doctors qualified as OHC irrespective of the training routes undertaken to qualify in occupational medicine. The introduction of this norm could be an important turning point in postgraduate medical education in Italy, paving the way for flexible specialist training programs allowing doctors to qualify in any specialist branch of medicine on the basis of the professional competences factually possessed. The professional skills required to practice occupational medicine (or any other medical discipline) could be acquired also outside formal university schemes, even abroad. For instance, a doctor able to demonstrate some of the skills expected from an OHC, having acquired them in another European country, should be given the opportunity to integrate and complete the rest of the training in Italy, eventually matching the same profile of a colleague fully trained within a specialist program of an Italian university (4).

Although focusing on a domestic Italian controversy on postgraduate medical training, the several points discussed above have important international implications across Europe, considering the difficulties but also the need of harmonizing professional education within a framework of gradual political and social integration between EU member states.
